# Supplementary material for: Selecting fitted models under epistemic uncertainty using a stochastic process on quantile functions
Source: Nat Commun. 2025 Oct 23;16:9393. doi: 10.1038/s41467-025-64658-7 (PMC12549851; doi:10.1038/s41467-025-64658-7)
Supplement: Supplementary file 1 — Supplementary Information [file 41467_2025_64658_MOESM1_ESM.pdf]

Selecting fitted models under epistemic uncertainty using a stochastic process on quantile functions

# – Supplementary Information –

Supplementary Methods

Transitivity of B^{EMD} comparisons

Given three independent continuous random variables R_A, R_B and R_C, define the probabilities

$$\underbrace{P(R_A < R_B)}_{=:B_{AB}} \qquad \underbrace{P(R_B < R_C)}_{=:B_{BC}} \qquad \underbrace{P(R_C < R_A)}_{=:B_{CA}} .$$

For some threshold ϵ, we would like these to satisfy a transitivity relation of the form

$$\left.\begin{aligned} B_{AB} &> \epsilon \\ B_{BC} &> \epsilon \end{aligned}\right\} \Rightarrow B_{CA} < \epsilon , \tag{S1}$$

as this would reduce the required number of pairwise comparisons between models (see section Model discrepancy as a baseline for non-stationary replications, Table 1 and equation (17) in the main text).

It is known that equation (S1) does not hold for $\epsilon = \frac{1}{2}$; classic counterexamples in this case are non-transitive dice.[2] However, the set of probabilities $\mathcal{S} \coloneqq \{B_{AB}, B_{BC}, B_{CA}\}$ *does* satisfy a property known as *dice-transitivity*,[4] from which one can derive that equation (S1) holds when $\epsilon = \varphi^{-1}$, where φ is the golden ratio. This result appears as a comment below Theorem 3 in Baets & Meyer,[1] but to our knowledge has otherwise remained unknown. We provide a short self-contained derivation below, for the convenience of the reader.

The definition of dice-transitivity is obtained by substituting equation (9) of De Schuymer *et al.*[4] into equation (6) of the same reference. For our purposes we are interested in the resulting upper bound

$$\alpha - 1 \leq -\beta\gamma , \tag{S2}$$

where α, β, and γ are respectively the lowest, middle and highest value of $\mathcal{S}$. In other words, $\{\alpha, \beta, \gamma\} = \mathcal{S}$ and

$$\alpha \leq \beta \leq \gamma . \tag{S3}$$

Suppose that, as given in equation (17), we have

$$B_{AB} > \varphi^{-1} \quad \text{and} \quad B_{BC} > \varphi^{-1} . \tag{S4}$$

We wish to use equation (S2) to establish an upper bound on B_{CA}. We do not know a priori how the probabilities are ordered, so we consider the six possible cases:

B_{AB}	B_{BC}	B_{CA}
α	β	γ
β	α	γ
α	γ	β
γ	α	β
β	γ	α
γ	β	α

(S5)

Cases $\alpha\beta\gamma$ and $\beta\alpha\gamma$. The assumptions of equation (S4) translate to $\alpha > \varphi^{-1}$ and $\beta > \varphi^{-1}$. We seek a bound on γ. Rearranging equation (S2), we then have

$$\gamma \leq \frac{1-\alpha}{\beta} < \frac{1-\varphi^{-1}}{\varphi^{-1}} = \frac{-1+\sqrt{5}}{2} = \varphi^{-1} , \tag{S6}$$

which contradicts equation (S3).

Cases $\alpha\gamma\beta$ and $\gamma\alpha\beta$. The argument is exactly analogous, except that we seek a bound on β. We get

$$\beta \leq \frac{1-\alpha}{\gamma} < \frac{1-\varphi^{-1}}{\varphi^{-1}} = \varphi^{-1} , \tag{S7}$$

which again contradicts equation (S3).

Therefore the only possible cases are $\beta\gamma\alpha$ and $\gamma\beta\alpha$, which means that B_{CA} must be the smallest of the three probabilities. These two final cases provide the upper bound on B_{CA}:

Cases $\beta\gamma\alpha$ and $\gamma\beta\alpha$. We seek a bound on α. Rearranging equation (S2) one more time yields

$$\alpha \leq 1 - \beta\gamma < 1 - \varphi^{-2} = \varphi^{-1} . \tag{S8}$$

Thus equation (S1) holds for $\epsilon = \varphi^{-1}$. More generally, we have $\alpha = B_{CA}$ whenever both B_{AB} and B_{BC} are greater than φ^{-1}. In this case equation (S8) implies

$$B_{AC} = 1 - B_{CA} > 1 - (1 - B_{AB}B_{BC}) = B_{AB}B_{BC} , \tag{S9}$$

and therefore

$$\left.\begin{aligned} B_{AB} &> \varphi^{-1} \\ B_{BC} &> \varphi^{-1} \end{aligned}\right\} \Rightarrow B_{AC} > B_{AB}B_{BC} . \tag{S10}$$

Equation (17) given in the main text is a special case of equation (S10).

Supplementary Results

Additional calibration curves for the neuron model

In Calibrating and validating the B^{EMD}, we considered only the effect of varying the external input strength (σ_i) on the

calibration curves for the Prinz model. Fig. 1 is an extended version of Fig. 6, where all 48 proposed epistemic distributions are plotted.

Sensitivity factor c can shift R-distributions

The primary effect of the sensitivity parameter c in equations (16) and (27) is to increase the spread of R-distributions, and thus their overlap. However, in contrast to the case where we simply add additive noise (e.g. the noise η in equation (S26)), the parameter c can also affect the shape of R-distributions, and in particular can change the relative distance between their centres. This is illustrated in Supplementary Fig. 2.

This phenomenon is one reason why the calibration curves actually change shape when we change c (c.f. Supplementary Fig. 1 and 4), and therefore that it is worthwhile to pin down an appropriate value for c. It is best understood as an effect of the additional constraints on $\mathfrak{Q}$: that realisations be monotone, integrable, and non-accumulating. These can change the shape of the $\mathfrak{Q}$ distribution, beyond the simple linear spread around q^* defined by 27.

This underscores also the importance of considering those constraints to correctly sample the space of experimental variations.

Comparison of selection criteria with inconclusive data

A key motivation underlying the B^{EMD} was to define a selection criterion which provides reliable estimates of its uncertainty, in particular for large test datasets (thousands of samples or more): these are commonly produced by experiments and yet produce pathologies with many of the common statistical methods of model selection. In short, a criterion can both over and underestimate the strength of the evidence in favour of one model.

We illustrate this phenomenon in Supplementary Table 1, with a set of comparisons meant to complement those of Fig. 8 in the main text and which explore the effect of misspecification in greater detail. We use 18 different variations of data generated with equation (38): three levels of noise s (ranging from high, 2^{12}, to low 2^{20} [$\mathrm{m}^2 \cdot \mathrm{nm} \cdot \mathrm{photons} \cdot \mathrm{sr} \cdot \mathrm{kW}^{-1}$]), three dataset sizes L (2^9, 2^{12}, and 2^{15}) and two wavelength ranges (20–1000 μm and 15–30 μm). Datasets with high noise and long wavelengths provide almost no discriminatory information: the two model predictions are almost the same, and any discrepancy between them is dwarfed by the amount of noise. At the other end, the low noise, short wavelength datasets are at the threshold between moderate and strong evidence in favour of $\mathcal{M}_{\mathrm{P}}$—in this regime the better model can already be identified by eye.

If Fig. 8 in the main text compares criteria on their terms, on a type of problem they were designed to tackle, Supplementary Table 1 explores what happens in a more challenging situation with misspecified models and an ambiguous selection problem. As with Fig. 8, all comparisons are done on the basis of a single test dataset, so replication uncertainty does not play a role.

In addition to the B^{EMD} and the risk (B^R), Supplementary Table 1 includes criteria based on the *likelihood ratio* (B^l), the *Bayes factor* (B^{Bayes})[5,7] and the *expected log predictive density* (elpd) (B^{elpd}).[8,5] We note that because $\mathcal{M}_{\mathrm{P}}$ and $\mathcal{M}_{\mathrm{RJ}}$ have the same number of parameters, the likelihood ratio is also equivalent to the *Akaike Information Criterion* (AIC), up to a factor of 2.[5]

To allow for comparability, for each criterion we report the log probability ratio; i.e. "model A is B^C_{AB} times more probable than model B." Conceptually, if $P(A)$ is the "probability of model A" and $P(B)$ the "probability of model B", then a criterion corresponds to

$$B^C_{AB} = \frac{P(A)}{P(B)} \quad \leftrightarrow \quad \log_{10} B^C_{AB} = \log_{10} P(A) - \log_{10} P(B)\,. \tag{S11}$$

This is the typical form for Bayes' factors, and we use Jeffreys' scale[6,7] to interpret values in Supplementary Table 1: values near 0, ±1 and ±2 respectively correspond to inconclusive, weak, and strong evidence.

Although only Bayes factors are typically reported in this form, most information criteria are defined in terms of log probabilities; the difference between the criteria of two models (the sign of which determines which model is selected) can be identified with the difference $\log_{10} P(A) - \log_{10} P(B)$ in equation (S11).

The main exception is the EMD criterion, which is defined in terms of a probability $P(R_A < R_B)$ rather than separate probabilities for A and B. To express it as a probability ratio, we define the "underbar" quantity, obtained by applying a logit transformation to $B^{\mathrm{EMD}}_{AB;c}$:

$$\log_{10} \underline{B}^{\mathrm{EMD}}_{AB;c} := \log_{10} B^{\mathrm{EMD}}_{AB;c} - \log_{10}\bigl(1 - B^{\mathrm{EMD}}_{AB;c}\bigr)\,. \tag{S12}$$

Values of $\underline{B}^{\mathrm{EMD}}_{AB;c}$ are therefore not directly comparable with those of $B^{\mathrm{EMD}}_{AB;c}$ reported elsewhere in this paper. In Supplementary Table 1, the $\log_{10} \underline{B}^{\mathrm{EMD}}_{AB;c}$ ranges from $-\infty$ to ∞, with values $\pm\infty$ indicating that the two R-distributions have zero overlap.

As a comparison, we also define $\underline{B}^R$, to show what happens if we simply compare the true risk:

$$\log_{10} \underline{B}^R_{AB} := (-R_A + R_B) \;/\; \log 10\,. \tag{S13}$$

The division by log 10 converts the basis of the logarithms from e to 10.

To interpret the results summarised in Supplementary Table 1, we highlight two types of undesirable behaviour:

Lack of saturation There should always come a point where enough data have been collected, and simply enlarging the dataset with samples from the same distribution does not provide more information. In the table therefore we want values to converge to a finite value as L increases.

Non-robustness The magnitude of a criterion should be an indicator of its robustness. If small changes in a dataset cause a criterion to flip from strongly negative to strongly positive, this suggests that its magnitude is not a good indication for the strength of the evidence.

If we are to interpret the values of criteria as relative probabilities, the global pattern we would expect to see in Supplementary Table 1 is that cells become progressively more blue as we

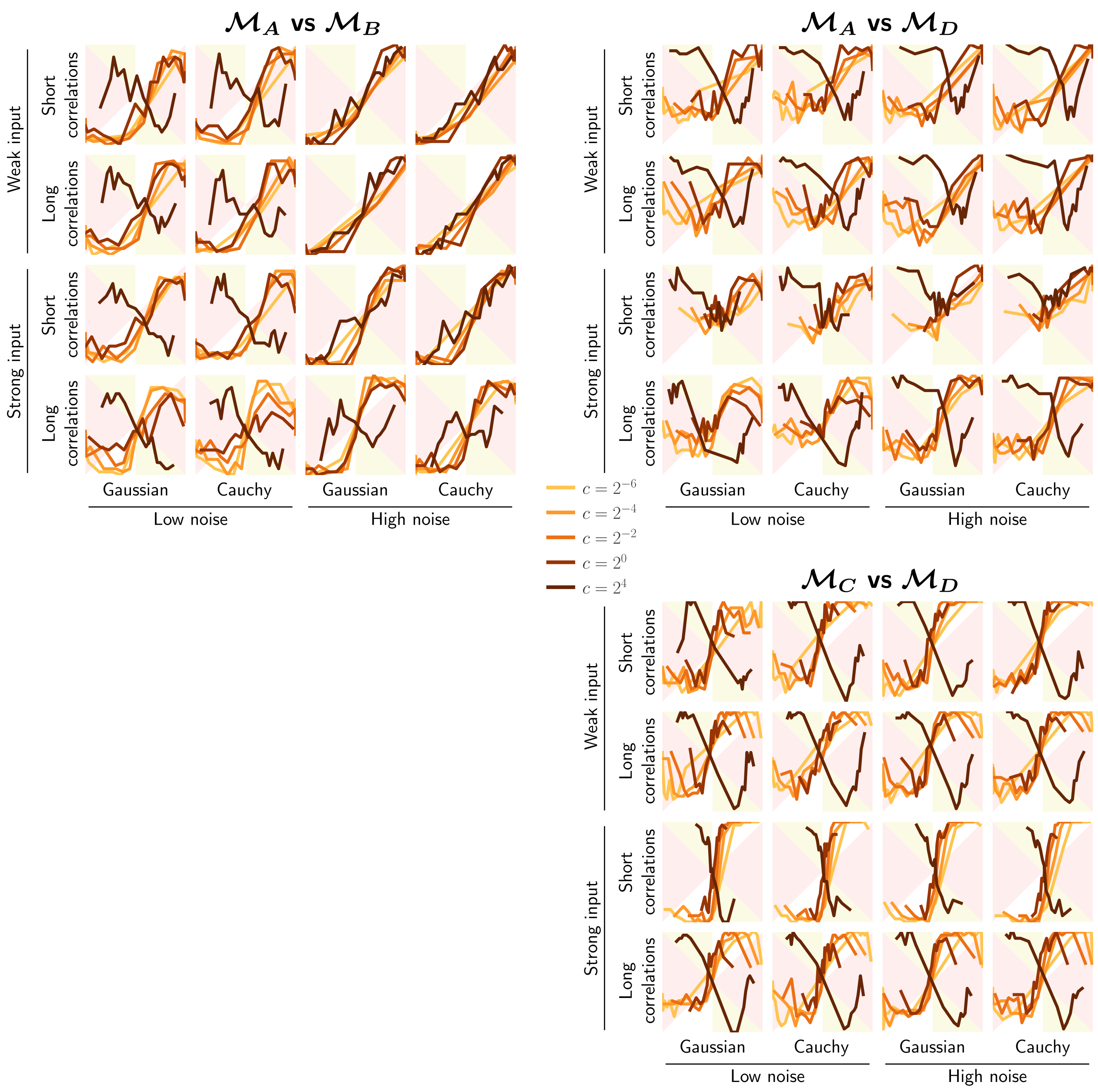

Supplementary Figure 1: **Additional calibration curves for the neuron model**, computed following the procedure described in section Calibrating and validating the B^{EMD} of the main text. Each curve summarizes 512 simulated experiments with datasets of size 4000. The regions depicted in red and yellow are those where equation (34) is violated.

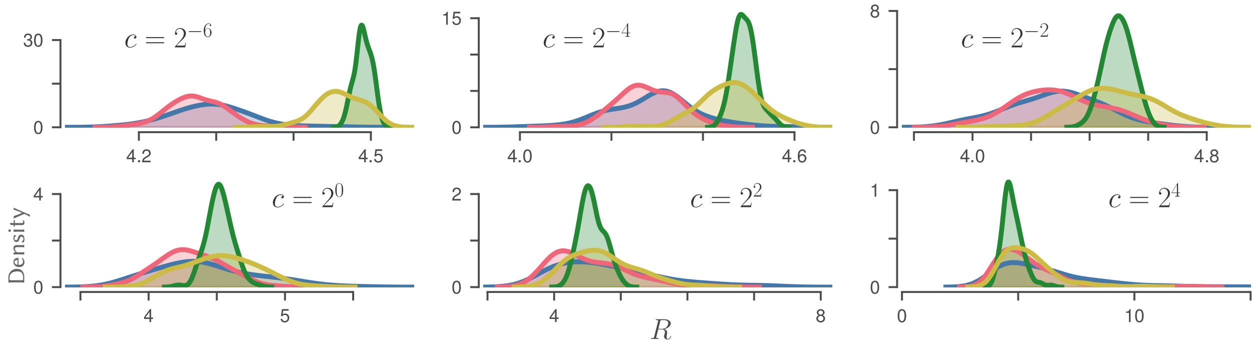

Supplementary Figure 2: R-distributions for the four candidate neuron models for different values of c.

go to the right (less noise, less model mismatch) and as we go down (more data, less ambiguous data).

Expressions used to compute selection criteria Conventions:

- $\mathcal{D}$: Training dataset used to fit the model.
- L: Number of data samples in $\mathcal{D}$.
- $(\lambda_i, \mathcal{B}_i)$: Sample in $\mathcal{D}$.
- $(\lambda'_j, \mathcal{B}'_j)$: Additional sample not in $\mathcal{D}$ (i.e. a test sample).
- $\hat{\sigma}, \hat{T}$: Estimates of σ and T obtained by maximizing the likelihood on $\mathcal{D}$.

EMD criterion

$$\log_{10} \underline{B}^{\mathrm{EMD}}_{AB;c} := \log_{10} B^{\mathrm{EMD}}_{AB;c} - \log_{10}\left(1 - B^{\mathrm{EMD}}_{AB;c}\right),$$

where $A = \mathcal{M}_{\mathrm{P}}$ and $B = \mathcal{M}_{\mathrm{RJ}}$ (repeated from equation (S12)).

Loss function As we did for the neuron model, we define the loss of a point $(\lambda_i, \mathcal{B}_i)$ as the negative log likelihood given that data point. Since the candidate models assume Gaussian noise (equation (39)), this is simply

$$\begin{aligned} Q_a(\mathcal{B}_i \mid \lambda_i, \sigma, T) &= -\log p\left(\mathcal{B} \;\middle|\; \mathcal{B}_a(\lambda; T), \sigma\right) \\ &= \log\sqrt{2\pi}\sigma + \frac{(\mathcal{B} - \mathcal{B}_a(\lambda;T))^2}{2\sigma^2} . \end{aligned} \tag{S14}$$

Here the subscript a is used to indicate whether we use predictions from the Rayleigh-Jeans (equation (36)) or Planck (equation (37)) model.

Log likelihood function Since we defined the loss as the negative log likelihood, one way to express the log likelihood for the whole dataset is simply as the negative total loss:

$$\ell_a(\sigma, T) := \sum_{i=1}^{L} -Q_a(\mathcal{B}_i \mid \lambda_i, \sigma, T) . \tag{S15}$$

Risk The risk is the expectation of the loss under the true model, so we have

$$R_a := \left\langle Q_a\left(\mathcal{B}'_j \mid \lambda'_j, \hat{\sigma}_a, \hat{T}_a\right)\right\rangle_{\lambda'_j, \mathcal{B}'_j \sim \mathcal{M}_{\mathrm{true}}}, \tag{S16}$$

$$\log_{10} \underline{B}^R := \left(-R_{\mathrm{P}} + R_{\mathrm{RJ}}\right) / \log 10 , \quad \text{(repeated from (S13))}$$

where $\lambda'_j, \mathcal{B}'_j \sim \mathcal{M}_{\mathrm{true}}$ indicates that λ'_j and $\mathcal{B}'_j$ are random variables with the same probability as the data.

Since in this case we know $\mathcal{M}_{\mathrm{true}}$, the expectation can be computed exactly. We did this by generating a very large number of data samples ($L = 2^{12}$) and computing the empirical average of the loss:

$$R_a \approx -\frac{1}{L} \ell_a(\hat{\sigma}_a, \hat{T}_a) . \tag{S17}$$

Bayesian prior The Bayesian calculations require a prior on the parameters σ and T. We used a simple prior which factorizes into two independent distributions:

$$\begin{aligned} \pi\left(\log_2 \sigma\right) &\sim \mathrm{Unif}\left(\left[\log_2 2^9, \log_2 2^{14}\right]\right), \\ \pi\left(\log_2 T\right) &\sim \mathrm{Unif}\left(\left[\log_2 1000, \log_2 5000\right]\right). \end{aligned} \tag{S18}$$

Here T is expressed in Kelvin and σ has units $\mathrm{kW} \cdot \mathrm{m}^{-2} \cdot \mathrm{nm}^{-1} \cdot \mathrm{sr}^{-1}$. We chose log uniform distributions because these are more appropriate for parameters which are strictly positive and which can span multiple scales: the logarithmic scaling captures the fact that the difference between 1000 K and 1001 K is more significant than the difference between 5000 K and 5001 K. Likewise for differences in the parameter σ at opposite ends of its range.

Expected log pointwise posterior predictive density (elpd)

$$\mathrm{elpd}_a := \left\langle \log_{10} p\left(\lambda'_j, \mathcal{B}'_j \mid a, \mathcal{D}\right)\right\rangle_{\lambda'_j, \mathcal{B}'_j \sim \mathcal{M}_{\mathrm{true}}} \tag{S19}$$

$$\log_{10} B^{\mathrm{elpd}} := \mathrm{elpd}_{\mathrm{P}} - \mathrm{elpd}_{\mathrm{RL}} . \tag{S20}$$

Note p in equation (S19) is a posterior density, and therefore evaluating it involves integrating over the prior:

$$\begin{aligned} &p\left(\lambda'_j, \mathcal{B}'_j \mid a, \mathcal{D}\right) \\ &= \iint d\sigma dT\, \pi_\sigma(\sigma)\pi_T(T)\, p\left(\lambda'_j, \mathcal{B}'_j \mid a, \mathcal{D}, \sigma, T\right). \end{aligned} \tag{S21}$$

Relative likelihood and AIC The relative likelihood is given by

$$\log_{10} B^l := \left(\ell_{\mathrm{P}}(\hat{\sigma}_{\mathrm{P}}, \hat{T}_{\mathrm{P}}) - \ell_{\mathrm{RL}}(\hat{\sigma}_{\mathrm{P}}, \hat{T}_{\mathrm{P}})\right) / \log 10 , \tag{S22}$$

while the difference between the AIC criteria of both models is (we use here the fact that both models have the same number of parameters)

$$\Delta AIC := 2\ell_{\mathrm{P}}(\hat{\sigma}_{\mathrm{P}}, \hat{T}_{\mathrm{P}}) - 2\ell_{\mathrm{RL}}(\hat{\sigma}_{\mathrm{P}}, \hat{T}_{\mathrm{P}}) . \tag{S23}$$

Since the two are equivalent up to a factor $2 \log 10$, the trends we see with the likelihood ratio therefore also occur with the AIC.

(The factor 2 in the AIC is meant to allow it to be interpreted—under certain assumptions—as a draw from a χ^2 distribution. This correspondence however is not required when interpreting Supplementary Table 1.)

Model evidence The model evidence is used to compute the Bayes factors. It is the expectation of the likelihood of the data—$\mathcal{D} = \{\lambda_i, \mathcal{B}_i\}_{i=1}^L$—under the prior for T and σ:

$$\begin{aligned} \mathcal{E}_a &:= \iint d\sigma dT\, \pi_\sigma(\sigma)\pi_T(T)\, p\left(\{\lambda_i, \mathcal{B}_i\}_{i=1}^L \mid a, \sigma, T\right) \\ &= \iint d\sigma dT\, \pi_\sigma(\sigma)\pi_T(T)\ell_a(\sigma, T) . \end{aligned} \tag{S24}$$

The likelihood $p\left(\{\lambda_i, \mathcal{B}_i\}_{i=1}^L \mid a, \sigma, T\right)$ is given by the Gaussian observation model.

Bayes factor

$$\log_{10} B^B := \log_{10} \mathcal{E}_{\mathrm{P}} - \log_{10} \mathcal{E}_{\mathrm{RL}} . \tag{S25}$$

Supplementary Table 1: **Comparison of different model selection criteria** for variations of the datasets shown in Fig. 7. Criteria compare the Planck model ($\mathcal{M}_{\mathrm{P}}$) against the Rayleigh-Jeans model ($\mathcal{M}_{\mathrm{RJ}}$) and are evaluated for different dataset sizes (L), different levels of noise (s) and different wavelength windows (λ). To allow for comparisons, all criteria have been transformed into ratios of probabilities. **Values reported are the** $\log_{10}$ **of those ratios.** Positive (blue shaded) values indicate evidence in favour of the Planck model, while negative (red shaded) values indicate the converse. For example, a value of +1 is interpreted as $\mathcal{M}_{\mathrm{P}}$ being 10 times more likely than $\mathcal{M}_{\mathrm{RJ}}$, while a value of -2 would suggest that $\mathcal{M}_{\mathrm{P}}$ is 100 times *less* likely than $\mathcal{M}_{\mathrm{RJ}}$. Noise levels are reported in two ways: s is the actual value used to generate the data, while "rel. σ" reports the resulting standard deviation as a fraction of the maximum radiance within the data window. The 20–1000 µm window for λ stretches into the far infrared range, where the two models are nearly indistinguishable; hence higher positive values are expected for zero bias, low noise, and the 15–30 µm window. As in Fig. 7, calculations were done for both positive and null bias conditions (resp. ($\mathcal{B}_0 > 0$ and $\mathcal{B}_0 = 0$); the former emulates a situation where neither model can fit the data perfectly. Expressions for all criteria are given in the supplementary text. For the $\underline{B}^{\mathrm{EMD}}_{\mathrm{P,RJ}}$ criteria, we used $c = 0.5$. Although this is the same value as we used for neuron model, it was determined through a separate calibration with different epistemic distributions.

			$\mathcal{B}_0 > 0$			$\mathcal{B}_0 = 0$		
		s	2^{12}	2^{16}	2^{20}	2^{12}	2^{16}	2^{20}
		rel. σ	35%	9%	2%	36%	9%	2%
Criterion	λ (μm)	L						
$B^{l}_{\mathrm{P,RJ}}$	20–1000	512	−0.72	−0.74	5.64	−0.72	−0.82	4.62
		4096	0.64	−0.41	14.49	0.62	−1.05	7.94
		32768	−3.02	10.33	40.41	−3.23	8.11	−8.50
	15–30	512	0.06	−0.35	3.27	0.06	−0.39	2.61
		4096	0.52	3.86	52.20	0.49	3.53	47.12
		32768	3.04	23.02	389.62	2.87	20.33	348.85
$B^{\mathrm{Bayes}}_{\mathrm{P,RJ};\pi}$	20–1000	512	0.02	−0.01	0.02	−0.06	0.04	-6×10^{-3}
		4096	0.06	−0.03	−0.02	0.04	−0.03	0.07
		32768	−0.05	−0.05	0.04	0.03	−0.03	−0.02
	15–30	512	-6×10^{-3}	−0.03	0.01	−0.02	-7×10^{-3}	−0.05
		4096	-4×10^{-3}	-5×10^{-3}	0.04	−0.04	−0.06	−0.01
		32768	0.02	0.06	−0.05	9×10^{-3}	0.05	−0.01
$B^{\mathrm{elpd}}_{\mathrm{P,RJ};\pi}$	20–1000	512	-4×10^{-3}	3×10^{-5}	3×10^{-4}	2×10^{-4}	2×10^{-3}	5×10^{-4}
		4096	-9×10^{-4}	1×10^{-3}	-4×10^{-3}	-3×10^{-3}	-3×10^{-3}	7×10^{-4}
		32768	2×10^{-4}	2×10^{-3}	-2×10^{-3}	1×10^{-5}	-7×10^{-5}	-2×10^{-3}
	15–30	512	-4×10^{-3}	-2×10^{-3}	-2×10^{-3}	6×10^{-4}	2×10^{-3}	-4×10^{-4}
		4096	-3×10^{-3}	1×10^{-3}	6×10^{-4}	6×10^{-4}	-8×10^{-4}	-2×10^{-3}
		32768	6×10^{-4}	-2×10^{-4}	-1×10^{-3}	-2×10^{-3}	-2×10^{-3}	-1×10^{-3}
$\underline{B}^{R}_{\mathrm{P,RJ}}$	20–1000	512	2×10^{-5}	-5×10^{-4}	5×10^{-3}	2×10^{-5}	-7×10^{-4}	4×10^{-3}
		4096	2×10^{-5}	-5×10^{-4}	5×10^{-3}	2×10^{-5}	-7×10^{-4}	4×10^{-3}
		32768	2×10^{-5}	-5×10^{-4}	5×10^{-3}	2×10^{-5}	-7×10^{-4}	4×10^{-3}
	15–30	512	8×10^{-5}	6×10^{-4}	0.01	8×10^{-5}	6×10^{-4}	9×10^{-3}
		4096	8×10^{-5}	6×10^{-4}	0.01	8×10^{-5}	6×10^{-4}	9×10^{-3}
		32768	8×10^{-5}	6×10^{-4}	0.01	8×10^{-5}	6×10^{-4}	9×10^{-3}
$\underline{B}^{\mathrm{EMD}}_{\mathrm{P,RJ}}$	20–1000	512	−0.04	0.11	0.33	−0.07	0.12	0.41
		4096	−0.01	0.07	0.34	−0.01	0.07	0.39
		32768	-1×10^{-3}	−0.04	0.26	-1×10^{-2}	−0.05	0.29
	15–30	512	−0.01	0.31	3.08	0.05	0.34	3.50
		4096	−0.03	0.40	1.87	−0.02	0.39	1.80
		32768	−0.05	0.24	1.74	−0.04	0.25	1.66

Supplementary Discussion

Other forms of uncertainty

As we say in the main text, there are two main sources of epistemic uncertainty on an estimate of the risk: limited number of samples and variability in the replication process. This work focusses on the estimating replication uncertainty, and numerically studying its effect as a function of sample size. We have eschewed a formal treatment of sample size effects, since this is a well-studied problem and good estimation procedures already exist.

For example, a bootstrap procedure can be used to estimate the uncertainty on a statistic (here the risk R) from a single dataset $\mathcal{D}$, by recomputing the statistic on multiple surrogate datasets obtained by resampling $\mathcal{D}$ with replacement (Supplementary Fig. 3**b**). Alternatively, if we have access to good candidate models, we can use those models as simulators to generate multiple synthetic datasets (Supplementary Fig. 3**c**). The distribution of risks over those datasets is then a direct estimate of its uncertainty due to finite samples.

In the limit of infinite data, both of these methods produce a risk "distribution" which collapses onto a precise value, independent of any discrepancy between model and true data-generating process. In contrast, that discrepancy defines the spread of risk distributions in Fig. 3, which do not collapse when $L \to \infty$.

Aleatoric uncertainty also does not vanish in the large L limit, but manifests differently. It sets a lower bound on the spread of pointwise losses a model can achieve (Supplementary Fig. 3**a**). In terms of our formalism therefore:

- **aleatoric uncertainty** determines the shape of the PPFs q^* and $\tilde{q}$,
- **epistemic uncertainty (due to finite samples)** is the statistical uncertainty on those shapes, and
- **epistemic uncertainty (across replicates)** is the propensity of the PPF to change when the experiment is replicated.

Both forms of epistemic uncertainty can contribute uncertainty on the risk, i.e. increase the spread of the R-distribution, but in the $L \to \infty$ limit only the effect of replications remains. Changes to aleatoric uncertainty will shift R-distributions along the R axis, but do not directly contribute epistemic uncertainty.

Flexibility in selecting c

An important property of our approach is that sensitivity parameter c does not need to be tuned to a precise value, but can lie within a range; either $c \in [2^{-4}, 2^{0}]$ for the neuron models of Fig. 6, or $c \in [2^{-6}, 2^{3}]$ for the Planck and Rayleigh-Jeans models of Fig. 9. This does not mean that the value of $B^{\mathrm{EMD}}_{ab;c}$ itself is insensitive to c: for fixed data, a larger c will generally bring $B^{\mathrm{EMD}}_{ab;c}$ closer to 50%. But the probability bound given by $B^{\mathrm{EMD}}_{ab;c}$ remains correct for all c within that range.

For example, we compute $B^{\mathrm{EMD}}_{\mathrm{P,RJ};c}$ to be 80% when $c = 2^{-3}$, versus 60% when $c = 2^{0}$. This means that if we fix $c = 2^{-3}$ (resp. $c = 2^{0}$), among all experiments which yield $B^{\mathrm{EMD}}_{\mathrm{P,RJ};c} = 80\%$ (resp. $B^{\mathrm{EMD}}_{\mathrm{P,RJ};c} = 60\%$), in at least 80% (resp. 60%) of them model $\mathcal{M}_{\mathrm{P}}$ will have lower true risk than $\mathcal{M}_{\mathrm{RJ}}$.

More generally, a $B^{\mathrm{EMD}}_{ab;c} = B$ (with $0.5 < B \leq 1$) states that, of all the experiments where the calculation of $B^{\mathrm{EMD}}_{ab;c}$ is equal to B, at least a fraction B of those will have $R_a < R_b$. In this respect the B^{EMD} exhibits some similarities with a confidence interval, in that it is interpreted in terms of *replications under a fixed computational procedure*: in the former case we have a fixed procedure for calculating $B^{\mathrm{EMD}}_{ab;c}$ given c, while in the latter case we have a fixed procedure for calculating the confidence interval given a confidence level. A key difference however is that with the B^{EMD}, the interpretation requires conditioning on the outcome of the calculation.

Of course the range of valid c values will depend on the variety of epistemic distributions, and cannot be guaranteed. In general, the larger the variety, the more difficult one can expect it to be to find a c which is valid in all conditions. We can however anticipate a few strategies which might increase the range of valid c values and otherwise improve the ability of the B^{EMD} criterion to discriminate between models:

Increased rejection threshold Increasing the rejection threshold ϵ in equation (15) can be a simple way to add a safety margin to the B^{EMD} criterion, at the cost of some statistical power, to account for small violations of equation (35).

Multi-step comparisons Initially, the large number of candidate models may force the selection of a larger (i.e. more conservative) c. After using this c to reject some of the candidates, it may be possible to reduce the value of c, thus increasing the discriminatory power and possibly further reducing the remaining pool of candidates.

Improved experimental control If one can improve the reproducibility across experiments, the epistemic distributions used for calibration can correspondingly be made tighter. This makes it easier to find a c which works in all experimental conditions, since there are overall fewer conditions to satisfy.

Domain-informed loss function A loss function designed with knowledge of the domain or target application can ignore irrelevant differences between models. In addition to improving the relevance of comparisons, this tends to make the risk a smoother function of experimental parameters, which should make the B^{EMD} easier to calibrate.

Post hoc correction of B^{EMD} values As long as we select a c for which the $B^{\mathrm{epis}}_{AB;\Omega}\left(B^{\mathrm{EMD}}_{AB;c}\right)$ function of equation (54) is monotone, we can use the calibration curves themselves to interpret values of $B^{\mathrm{EMD}}_{AB;c}$ by looking to the $B^{\mathrm{epis}}_{AB;\Omega}$ to which they map. This approach could be used to improve discriminatory power of a conservative B^{EMD}, but also to correct it in regions where it is overconfident—assuming one has sufficient trust that the calibration curves are truly representative of experimental variations.

Comparing models directly with the loss distribution

To further illustrate the previous point, we consider an alternative comparison criterion which directly uses the distribution

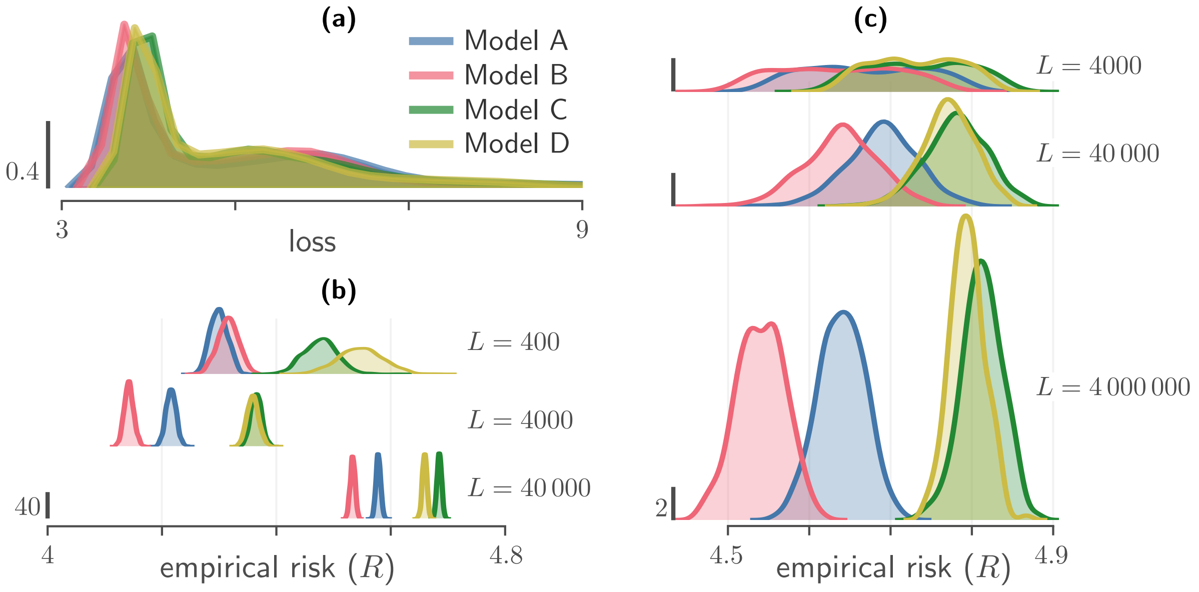

Supplementary Figure 3: **Aleatoric and finite-size uncertainty.** **a) Loss distribution** of individual data points—$\{Q_a(t_k, V^{\mathrm{LP}}(t_k; \mathcal{M}_{\mathrm{true}}))\}$—for the dataset and models shown in Fig. 2 and $a \in \{A, B, C, D\}$. For a model which predicts the data well, this is mostly determined by the aleatoric uncertainty. **b) Bootstrap estimate of finite-size uncertainty** on the risk, obtained using case resampling:[3] for each model, the set of losses was resampled 1200 times with replacement. Dataset and colours are the same as in (a); the same L data points are used for all models. **c) Synthetic estimate of finite-size uncertainty** on the risk, obtained by evaluating equation (10) on 400 different simulations of the candidate model (differing by the random seed). Here the same model is used for simulation and loss evaluation. Dataset sizes L determine the integration time, adjusted so all datasets contain the same number of spikes. All subpanels use the same vertical scale. **b–c)** The variance of the R-distributions, i.e. the uncertainty on R, goes to zero as L is increased. **a–c)** Colours indicate the model used for the loss. Probability densities were obtained by a kernel density estimate (KDE).

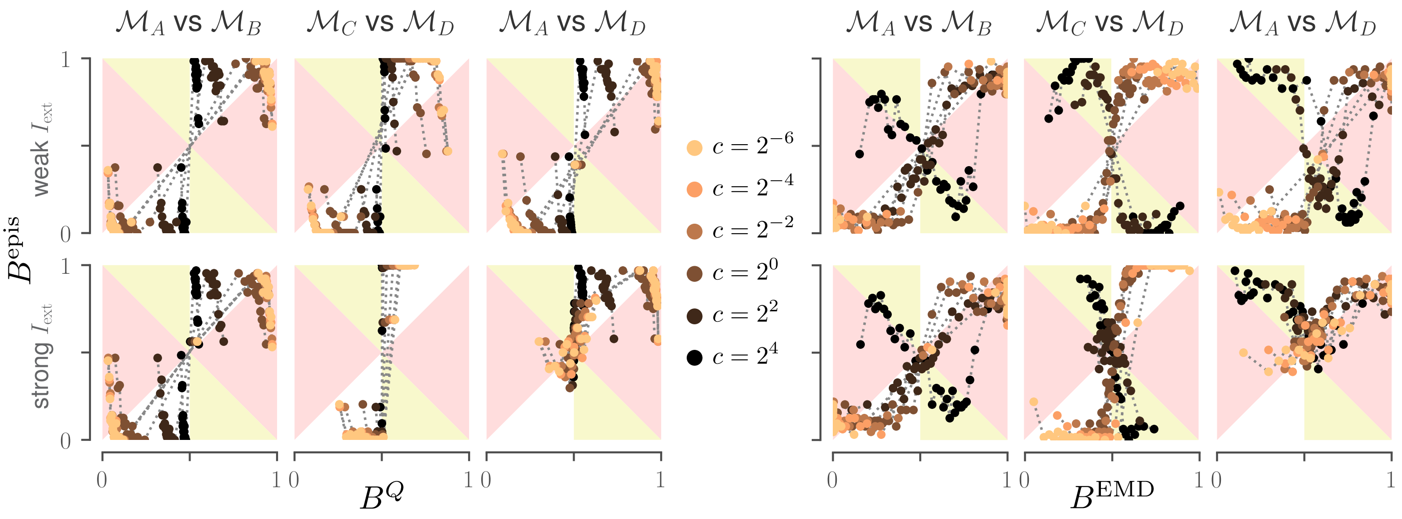

Supplementary Figure 4: **(a) Calibration experiments for the neuron model, using B^Q** (equation (S26)) instead of B^{EMD} (equation (16)) as a comparison criterion. To better show the distribution of experiments, each histogram bin (see Calibration experiments in the Methods) is represented as a point. **(b)** Same curves as in Fig. 6, this time with bins presented as points to ease comparison with (a). Compared to (a), points are more uniformly distributed along both the horizontal and vertical axes. All panels use the same set of sensitivity values (either c_Q or c), with colours as indicated in the central legend.

of losses Q (i.e. the distribution in Supplementary Fig. 3**a**) instead of the more complicated process $\mathfrak{Q}$ over PPFs with which we defined the B^{EMD}. To this end, let us define a B^Q criterion in analogy with equation (16):

$$\begin{aligned} B^Q_{ab;c_Q} &:= P(Q_a < Q_b + \eta)\,, \\ \eta &\sim \mathcal{N}(0, c_Q^2)\,. \end{aligned} \tag{S26}$$

The Gaussian noise η is added to allow us to adjust the sensitivity of the criterion, analogously to how c adjusts the sensitivity of the $B^{\mathrm{EMD}}_{ab;c}$ criterion. Both criteria thus have one free parameter, making the comparison relatively fair.

Note that in contrast to the B^{EMD}, the c_Q values in the B^Q are not dimensionless. Consequently, they would likely be less transferable between experimental contexts.

A possible argument in favour of using B^Q as a criterion is that increasing the amount of misspecification—for example by increasing the unmodelled bias $\mathcal{B}_0$ in equation (38)—will affect the distribution of losses in some way. So although the shape of the PPFs is mostly determined by aleatoric uncertainty (as evidenced by the similarity of the distributions in Supplementary Fig. 3**a**), one can expect them to also contain some information about the amount of misspecification—and thereby also the amount of epistemic uncertainty.

Nevertheless, as we see in 4 , the B^Q criterion is less effective for comparing models than the B^{EMD} in at least three ways (recall that the goal is not to find a criterion which accurately predicts the model with lowest risk, but one which accurately predicts the *uncertainty* on that risk, i.e. B^{epis}):

Reduced signal Only very few experiments on the main diagonal have B^{epis} values different from 0 or 1. The B^Q values therefore don't really inform about the epistemic uncertainty. Learning a monotone transformation from B^{EMD} or B^Q to B^{epis} is only possible if the curve is strictly monotone.

Increased anti-correlation between B^Q and B^{epis}: in five out of six comparisons between neuron models, we see strong anticorrelated tails at both ends of the curves, which dip all the way back to $B^{\mathrm{epis}} \approx 0.5$. This makes it harder to find a c_Q for which the the B^Q is actually informative, since the curve is not even injective. While there is some anticorelation also with the B^{EMD} (4**b**), it is much less pronounced and limited to comparisons with the worse models ($\mathcal{M}_C$ and $\mathcal{M}_D$).

The sensitivity parameter does not really help Increasing c_Q squeezes curves horizontally, bringing all B^Q values closer to 0.5, but does not change their shape nor reduce the length of anticorrelated tails at both ends. All curves therefore effectively provide the same information. In contrast, the c parameter has a much stronger effect on the shape of R-distributions (c.f. Supplementary Fig. 2).

These results suggest that an approach based only on distributions of the loss Q is likely to always struggle with disentangling epistemic from aleatoric uncertainty. It also has less information to work with: whereas B^{EMD} is parameterised by two functions, q^* and δ^{EMD}, B^Q is parameterised by only one, the density $p(Q)$. Moreover, $p(Q)$ and q^* contain the same information (they are the PDF and PPF of the same random variable), so the discrepancy function δ^{EMD} provides B^{EMD} with strictly more information—information which is also specifically designed to disentangle the effects of misspecification from aleatoric uncertainty.

Supplementary References

1. Baets, B. D. & Meyer, H. D. in *Perception-Based Data Mining and Decision Making in Economics and Finance* (eds Batyrshin, I., Kacprzyk, J., Sheremetov, L. & Zadeh, L. A.) 261–274 (Springer, Berlin, Heidelberg, 2007). ISBN: 978-3-540-36247-0. doi: 10.1007/978-3-540-36247-0_10.
2. Conrey, B., Gabbard, J., Grant, K., Liu, A. & Morrison, K. E. Intransitive Dice. *Mathematics Magazine* **89,** 133–143. ISSN: 0025-570X, 1930-0980. doi: 10.4169/math.mag.89.2.133 (Apr. 2016).
3. Davison, A. C. & Hinkley, D. V. *Bootstrap Methods and Their Application* ISBN: 978-0-521-57471-6. doi: 10.1017/CBO9780511802843 (Cambridge University Press, Cambridge, 1997).
4. De Schuymer, B., De Meyer, H. & De Baets, B. Cycle-Transitive Comparison of Independent Random Variables. *Journal of Multivariate Analysis* **96,** 352–373. ISSN: 0047-259X. doi: 10.1016/j.jmva.2004.10.011 (Oct. 2005).
5. Gelman, A. *et al. Bayesian Data Analysis* ISBN: 978-1-4398-9822-2 (CRC Press, Boca Raton, 2014).
6. Marco Taboga. in *Lectures on Probability Theory and Mathematical Statistics* Online appendix (Kindle Direct Publishing, 2021).
7. Trotta, R. Bayes in the Sky: Bayesian Inference and Model Selection in Cosmology. *Contemporary Physics* **49,** 71–104. ISSN: 0010-7514. doi: 10.1080/00107510802066753 (Mar. 2008).
8. Vehtari, A., Gelman, A. & Gabry, J. Practical Bayesian Model Evaluation Using Leave-One-out Cross-Validation and WAIC. *Statistics and Computing* **27,** 1413–1432. ISSN: 1573-1375. doi: 10.1007/s11222-016-9696-4 (Sept. 2017).
